# Design and Evaluation of Peer Supervision for Community Mental Health Workers: A Task-Shifting Strategy in Low-Resource Settings

**DOI:** 10.1007/s10597-023-01161-7

**Published:** 2023-09-07

**Authors:** Rekha Pallikkuth, T. Manoj Kumar, Claudia T. Dictus, J. F. G. Bunders-Aelen

**Affiliations:** 1 Department of Clinical Psychology, Mental Health Action Trust, Calicut, India; 2 Athena Institute, VU University Amsterdam, Amsterdam, The Netherlands

**Keywords:** Community mental health, Low- and middle-income countries, Peer supervision, Social cognitive theory, Lay health workers

## Abstract

The use of Lay Mental Health Workers (LMHWs) to tackle the treatment gap in low-resource settings is well established, and although they often receive training, the potential of proper supervision to improve outcomes remains untapped. Indeed, given the strain on expert resources, peer-supervision models based on supervisors’ seniority of work experience have significant potential especially in relation to community knowledge and embedding of LMHWs. This study summarizes the evaluation of a pilot program for peer supervision on the basis of Social Cognitive Theories of Self-Efficacy for LMHWs in Kerala, India. Two experienced LMHWs worked as supervisors for a total of 12 LMHWs over the course of a year. These participants were subsequently interviewed to analyze their experiences in order to evaluate the potential of peer supervision and distil relevant information to improve future training of LMHWs. The findings include improved performance and emotional support for the participants.

## Introduction

Mental disorders are a leading cause of disability and account for approximately 23% of all years lived with disability worldwide (Whiteford et al., 2017). In low-resource settings there are often significant treatment gaps, with 75% of those with a mental disorder never receiving proper care (Thornicroft et al., 2019). There is a need for services which are feasible, scalable, and sustainable in such contexts, especially given the critical shortages of financial and human resources (Davies & Lund, 2017; Patel et al., 2010). It has been argued that low- and middle-income countries (LMICs) would benefit from health models that are not over-reliant on specialized mental health professionals for interventions, for instance by employing Lay Mental Health Workers (LMHWs), to increase access to existing mental health services (Kola, 2020; Mutamba et al., 2013; World Health Organization, 2018).This approach, which has been developed to close the mental health gaps in LMICs, relates to the concept of task sharing, explained by Kemp et al., (2019, p. 150) as;

An arrangement in which generalists—non-specialist health professionals, lay workers, affected individuals, or informal caregivers—receive training and appropriate supervision by mental health specialists and screen for or diagnose mental disorders and treat or monitor people affected by them.

This approach has proven feasible and effective in helping patients with mental health problems in circumstances where there are few available resources (e.g., Kakuma et al., 2013). Yet studies also indicate a need for building LMHWs’ confidence and competence in their work with mental health patients (Herschell et al., 2010; Kohrt et al., 2018). For instance, Kemp et al. (2019) found that besides the need for proper training in basic skills, it is necessary to address proper supervision and mentorship to establish a continuous learning cycle, so that situations such as faulty diagnosis and treatment of patients can be corrected and positive behaviors can be reinforced through reflection and feedback cycles. One condition, evident from concerns about resource constraints, is that the supervisory model should not be too costly, and be relatively easy to apply. In this context, peer-supervision has been suggested as sustainable alternative to standard, hierarchical supervision (e.g., Hill et al., 2014). Understanding how to strengthen the needed confidence and competence in LMHWs requires a structured approach, for which Bandura’s model of ways to improve self-efficacy (individuals’ belief in their ability to perform a given task) presents a useful tool (1995). The general self-efficacy literature suggests that it is an effective predictor of performance (Bandura & Adams, 1977; Bandura et al., 1977; Bandura et al., 1980). More importantly, it suggests that self-efficacy predicts performance outcomes better for some areas, such as improving the quality of care, team work, administrative support, self-care and emotional support.

### Peer Supervision

Between the multiple and at times conflicting definitions of peer supervision identified in the literature, the main common feature is that it involves an approach in which the supervisor and supervisee are of similar hierarchical status or perceive themselves to be equals, which results in improved support, enhanced learning and problem solving (Aftab et al., 2018; Amanvermez et al., 2020, Bagonza et al., 2020). Different definitions hold that it should take place in a group setting or involve a reciprocal activity between two individuals, but there are also numerous instances in which one set of more experienced or high-performing peers is given training on how to provide support to others in the same position (Amanvermez et al., 2020; Bagonza et al., 2020; Hossain et al., 2021). Given that the main benefits of this approach are the empathy between peers and the reduced costs in low-resource settings, this research takes the position that peer supervision can be best defined as a supervisory or mentorship relationship between peers (perceived equals) in order to improve learning and development while keeping costs and feasibility manageable (Hill et al., 2014). Some studies experimenting with peer supervision have found it a cost-effective alternative to standard supervision (Chang & O’Hara, 2011; Crigler et al., 2013; Henry et al., 2016; Hill et al., 2014). In particular, it has been shown to result in greater commitment to work, and more creative problem solving (Crigler et al., 2013). Peer supervision does not require the presence of fully accredited experts, but relies instead on a supervision process that emphasizes critical and supportive feedback (mentoring), rather than direct monitoring of work (Basa, 2018). In the context of LMHWs, there may be particular challenges in relation to the supervisors’ self-esteem, given a tendency to undervalue experience-based knowledge, though this can likely be overcome through mutually supportive interactions.

Given the importance of supporting effective, feasible practices in low-resource settings that adapt to the local context, peer supervision is an essential aspect of discussion and development in this field of research. Peer supervision can take many forms—from group meetings for problem-solving to one-to-one observation and feedback where stronger or more senior peers can provide guidance (Crigler et al., 2013; Hill et al., 2014). Components of peer supervision differ depending on the specific study, but frequently include an emphasis on the quality of care, self-assessment, checklists and multi-level supervision (Rothwell et al., 2021).

Given the increasing use of LMHWs to provide health services, and the documented role that quality supervision can play in maintaining the performance and motivation of formal health workers and LMHWs, there is a critical need for knowledge about effective peer-supervision strategies and their implementation (Aftab et al., 2018; Assegaai & Schneider, 2019; Kok et al., 2017; Roberton et al., 2015; Schwarz et al., 2019). Some studies have examined the changes in self-efficacy during peer supervision, with improvement related to five areas: skills, process, handling patients’ difficult behavior, cultural competence, and awareness of ethical values (Aftab et al., 2018; Assegaai & Schneider, 2019; Schwarz et al., 2019).

### Peer Supervision in Culturally Diverse LIMC Contexts

Peer supervision has been found to facilitate reflection, sharing ideas, personal growth and self-care skills (Glassburn et al., 2019; O’Donovan et al., 2018; Posluns & Gall, 2019; Schumann et al., 2020; Tseng et al., 2019). Some studies show that there may be resistance in specific contexts relating to conflicts with organizational culture, fearing value-based judgments, lack availability of (or trust in) internal supervisors (Hill et al., 2014). For example, in one study in India, supervisors did not like solving problems in a participatory fashion as they preferred to maintain their hierarchical status (Haas, 2019). There has been some research on the use of peer supervision in the context of lay health workers in LMICs in particular. For instance, positive effects of peer supervision in task-shared settings have been found in studies in South Africa (Assegaai et al., 2019), Pakistan (Aftab et al., 2018), Uganda (Bagonza et al., 2020), Kenya (Hossain et al., 2021) and in Bangladesh, Indonesia and Mali (Hill et al., 2014). Though literature reviews have thus far found few articles of sufficient quality to draw broader conclusions, initial studies indicate the potential of this approach to address power imbalances and high costs, and a study by Kemp et al. (2019) working with multiple informants in these settings highlighted the promising potential of peer supervision. There is thus a need for further empirical evaluation of the implementation of peer-supervisory models in LMIC contexts (Haas, 2019). Peer supervision is a promising way to strengthen the function of LMHWs in the treatment of mental illness in low-resource settings on the basis of their practical knowledge, but only when conducted in a manner that fits the cultural setting, values and expectations (Haas, 2019).

The present research aims to understand the practical application of peer supervision in one community clinic in India in relation to how peer supervision might affect self-efficacy and the possible consequences for performance outcomes, in the hope that this might generate more insights for future application in similar settings. The study addresses three sub-goals: first, to reflect on the use and evaluation of peer supervision in the workplace; second, to identify what types of problems LMHWs encounter; and third, to determine what can be learned about the best way to respond, by analyzing the supervisory practices in relation to LMHWs’ role and functioning in their communities, and how these can also be used to improve the training and supervision practices in the future.

## Theoretical Framework

### Self-efficacy

Self-efficacy can be understood as people’s beliefs or judgments about their ability to accomplish a given goal or task (Bandura, 1995). It is also a recognized measure of development in the mental health field, has a positive influence on work-related performance, and consequently works as an outcome and developmental consideration for training mental health workers (Bandura et al., 1980; Larson & Daniels, 1998; Stankovic & Luthans, 1998). In addition, there is a substantial body of published research examining those who are undergoing training in mental health and their self-efficacy (e.g., Barbee et al., 2003; Cashwell & Dooley, 2001; Kozina et al., 2010; Melchert et al., 1996; Tang et al., 2004). There is, however, only limited research on LMHWs’ development of self-efficacy over time, based upon their experiences in a task-shared community mental health setting.

**Fig. 1:**
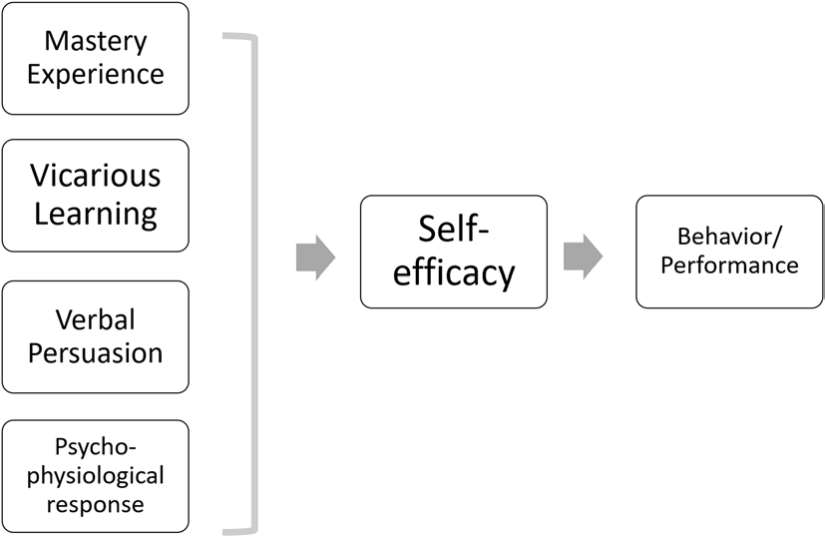
Bandura’s model of self-efficacy

The theoretical framework for this study is based on Bandura’s Social Cognitive Theory (SCT) (see Fig. 1). The research used Bandura’s four proposed aspects of self-efficacy development—experience of mastery, vicarious learning, verbal persuasion, and affective reaction/physiological state—as a lens through which to view the research findings (Bandura, 1986; Morrison & Lent, 2018). Bandura argues the most effective way to build self-efficacy is through (1) experiencing mastery of skills personally; (2) by witnessing demonstrations of competence by similar people; and/or (3) and/or being told by someone we trust that we have the ability to achieve our goals (Morrison & Lent, 2018). Finally, Bandura notes that it is harder for a person to feel assured of their ability to succeed when they feel weary and in a low mood (Morrison & Lent, 2018). This is especially true if these emotional and physiological states are perceived to be indicative of incompetence, vulnerability, or inability to achieve a goal. Given its representation of these diverse aspects, this theory can be used to describe the individual development of participants’ self-efficacy (Lent, 2016). Indeed, this construct of self-efficacy has for some years been mainstreamed into supervisory research (Lent et al., 1996; Lockwood et al., 2017; Mesrie et al., 2018; Morrison & Lent, 2018; Mullen et al., 2015). The need for personnel who are working in the field of mental health to feel confident in their ability to help patients effectively is crucial to the interpersonal experience and therapeutic alliance (Mesrie et al., 2018; Morrison & Lent, 2018).

**Fig. 2:**
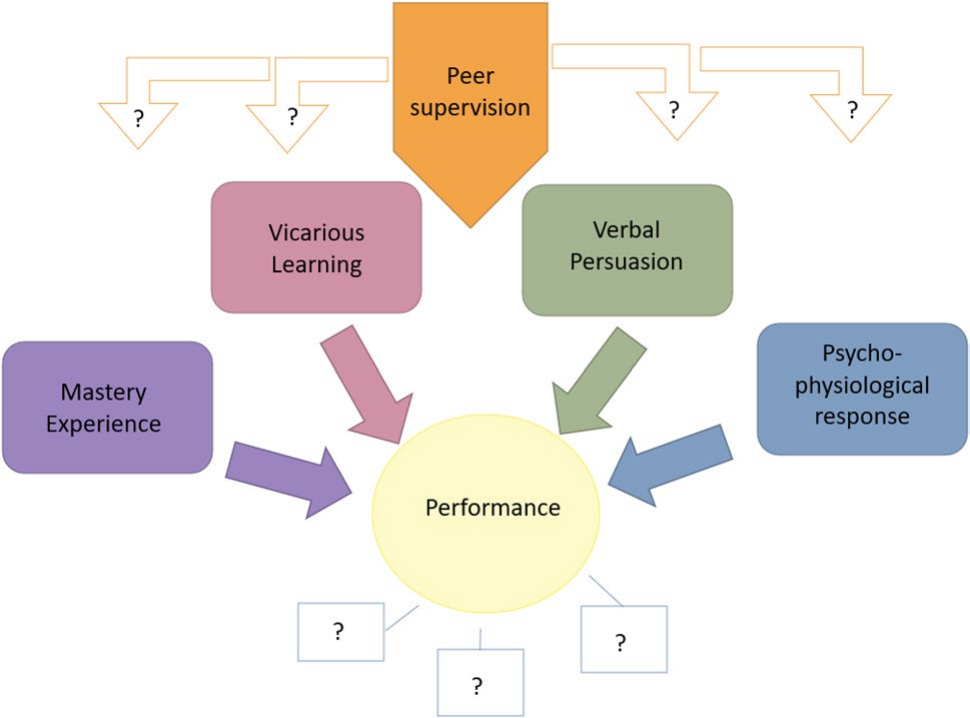
Theoretical framework

In order to apply this model, the research approach was rooted in full-time peer supervision (on-site or by phone) to allow for the managing, training, mentoring and monitoring of LMHWs, in order to build relationships and foster trust between community-based mental health services and the communities in which they exist. The primary research question relates to the supervision experiences of LMHWs as understood through Bandura’s analytical framework. It is anticipated that insights into the mechanisms might also help in developing future training modules for LMHWs. The integration of self-efficacy within the understanding of the impact of peer supervision on performance outcomes is set out in Fig. 2. It demonstrates the interest of this research in both the links between peer supervision and aspects of self-efficacy, and the particular types of performance outcomes that may be strengthened through peer supervision.

## Methods

### Research Setting

The current study was conducted in the context of Mental Health Action Trust (MHAT), a non-government organization (NGO) based in Kozhikode, in the Indian state of Kerala. MHAT provides free mental health services to economically disadvantaged people in several districts of Kerala. For 13 years, comprehensive multidisciplinary care has been provided via local partnerships with the health system and LMHWs. The LMHWs undertake the bulk of the community-based work and are central in the MHAT’s approach. Their roles range from screening and regular domiciliary monitoring of patients to providing group and individual psychosocial interventions, rehabilitation, and family-focused interventions. The study was conducted from August 2020 to April 2021 on peer supervision of LMHWs in a task-shared, recovery-oriented community mental health setting in rural Kerala.

### Supervisory Process

Peer supervisors were trained LMHWs who had undertaken 3 years of experiential training on supervision with a senior clinical psychologist with 30 years’ experience in clinical supervision, had completed an eight-week classroom training program and had had 6 years of supervised field work experience in community-based psychosocial interventions for severe mental illness via MHAT from 2013 to 2019. Supervisees consisted of the LMHWs with less than 4 years’ experience and who had not undergone supervised field work experience with MHAT. Peer supervision by trained LMHWs was offered in one-to-one individual sessions over the course of 12 months (August 2020 to April 2021). Supervisory discussions aiming to improve quality and provide support focused on (a) providing an opportunity for the experience of mastery; (b) providing information and knowledge through modeling and experience sharing; (c) providing feedback; (d) encouraging through persuasion; and (e) providing emotional support. The supervisors maintained written supervision records, and supervisees were encouraged to keep their own records as well.

The sessions were structured to provide an opportunity to observe supervisors’ activities, share their experiences, and provide constructive feedback and guidance in a supportive environment. Peer supervisors were expected to meet the people they were supervising every week for at least an hour. The peer supervisors met at community clinics once every 2 weeks, along with a weekly phone meeting. The observation took place in cases where supervisees felt stuck. The supervisory process was integrated within the working/learning environment on an ongoing basis as naturally as possible in relation to usual tasks and duties.

A facilitated, supervisor-led model was used, which affords supervisees the opportunity to share their experiences with their senior colleagues and receive feedback. One-to-one peer supervision in MHAT was provided for 12 LMHW staff members by two peer supervisors, each of whom met with six supervisees on a one-to-one basis. Supervision sessions took place in private and quiet work-based locations or by phone.

### Operationalization of Theoretical Framework

In order to link the theoretical understandings of self-efficacy with the concrete actions of supervision and the measurable outcomes related to performance in practical terms, this study developed an overview that connects the planned supervisory activities to potential effects on self-efficacy and performance (Table 1).

**Table 1:** Operationalization theoretical framework

HOW (process)		
Bandura’s sources of self-efficacy	Planned mode of induction through peer mentorship	Hypothesized effects on self-efficacy
1. Mastery experiences	Feedback on individual performance outcomes Positive feedback resulting in mastery experiences	Improved confidence through evidence of successes Improved skills through learning from mistakes
2. Vicarious experiences	Providing information and knowledge through modeling and experience sharing Observing peer supervisors making efforts and succeeding in their activities	Lessons from others’ experience Emotional development (‘If they can do it, I can do it as well’) Stronger sense of commitment to their activities
3. Verbal persuasion	Advice and encouragement from peer supervisor	Pushed to action and able to achieve further successes. Experience of overcoming challenges
4. Psycho-physiological response	Providing emotional support by peer supervisor Reflection on emotional states in front of peer supervisor	Quicker recovery from setbacks and disappointment Lessons on stress management and dealing with challenges

### Participants

Participants were recruited using purposive sampling. Before starting the supervision, participants were informed verbally and LMHWs were invited to participate in the study; they were also given two reminders about the interview. Each participant received a minimum of 12 supervisory sessions of an hour over a six-month period. Participants were assured that their participation was voluntary and that they could withdraw from the study with no negative effects on their employment. Peer supervision was offered to 12 LMHWs who consented.

### Data Collection

ALL participants completed a brief socio-demographic questionnaire before the interviews and qualitative data was gathered through semi-structured interviews with each participant. The interviews aimed to explore the participants’ perceptions of the peer-supervisory relationship in acquiring skills. Interviews were conducted between January and April 2021 and participants were also offered the option of telephone interviews to accommodate busy work schedules, geographical location, and pandemic-related social distancing. All interviews were audio-recorded and conducted by experienced researchers who were not known to the participants. Data was handled and stored according to the Mental Health Action Trust’s Data Protection Policy, ensuring confidentiality.

### Data Analysis

**Fig. 3:**
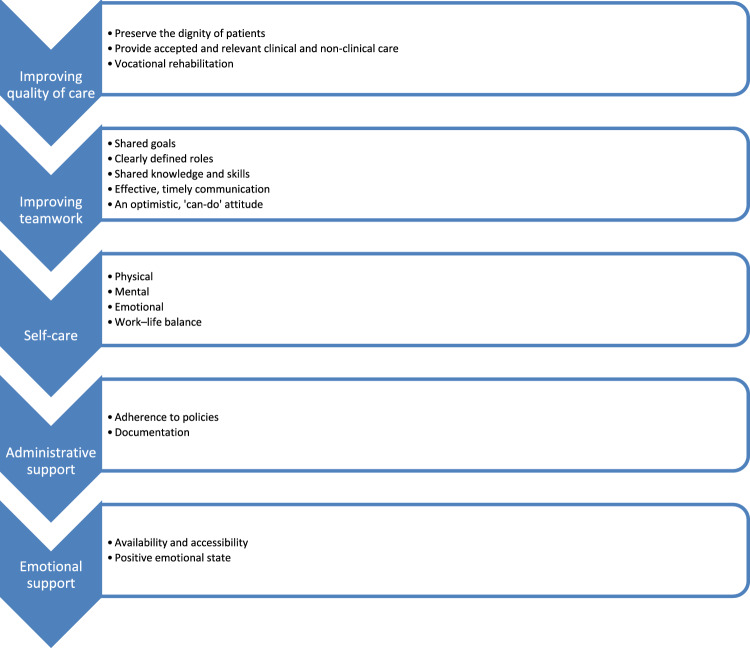
Overall performance outcome

The primary focus of the interview guide related to how the peer supervision went, how the supervisees perceived it and what effect it had, with emphasis on self-efficacy. Interviews were transcribed verbatim, omitting personal identifiers after which they were analyzed, first using deductive coding followed by inductive shifting. Initial analysis involved coding on the basis of the four sources of self-efficacy. However, when analyzing the effects of the sources of self-efficacy on participants, the research team noticed patterns emerging relating to particular roles or tasks the LMHWs performed as well as particular types of outcomes. These emergent patterns were consolidated into five themes, each with at least two sub-themes, within which the codes from the framework could be explored more precisely (See Fig. 3). The final codebook was applied to each interview, which was then cross-checked for accuracy.

Concerning the first theme, gaps in LMHWs’ skills and challenges they experienced became clear through those aspects of supervision they identified as having an impact. These challenges were then linked to the concept of quality care, where each of the three sub-sections identified have an impact on whether services affect the intended mental health outcomes. How LMHWs’ behavior can affect the dignity of clients, guidance on particular activities that affect quality of life, and the importance of vocational rehabilitation for empowerment emerged as three areas in which supervisory activities (categorized based on the framework) had an effect.

The second theme emerging from the data related to the complexities of working as part of a clinical team. Fostering healthy relationships between the various actors involved, and in particular navigating dynamics of authority and trust, was understandably identified as a challenging area where supervision was able to generate improvements. Sub-themes in this aspect of coding were related to the key factors for successful communication that the participants highlighted they had learned about. The setting of goals as a team, clearly dividing roles and responsibilities and exchanging knowledge in the team, communicating feedback to others in a timely manner, and a positive attitude emerged as qualifications for successful communication and team work.

The remaining three themes related to roles and aspects of the work of LMHWs beyond direct patient interactions and clinical work. The emergent quality of these themes was among the reasons for the choice to expand the analytical framework to consider inductive codes. Self-care as a pre-requisite for providing quality care was a novel concept for some participants, and the prioritization of one’s own health in order to provide care to others was an interesting lesson in self-efficacy. The fourth theme relating to the administrative tasks of LMWHs might not often be seen as a priority, but its importance became clear to participants in relation to the smooth functioning of the clinic through the supervisory process. Finally, the importance of the emotional support provided through the supervisory process for the participants’ increased wellbeing and functioning emerged with such force that it needed to be considered separately from the other underlying emotional dynamics that were observed within the other themes.

The application of these emergent themes as the primary analytical unit provided space to explore more specific examples of Bandura’s sources of self-efficacy within each of the sub-sections, where quotes could be coded using both the aspect of improved performance to which it related and which of the sources and strategies could be identified.

## Results

Participants’ characteristics are explained in the first part of this section. The remaining findings consist of an overview of the experience of peer supervision categorized under sub-headings related to codes from the analysis. After a brief discussion of participants’ characteristics, the results are structured in two main sections: performance outcomes with five sub-sections, as indicated in Fig. 3; and emotional support. The way participants experienced support and attained self-efficacy were analyzed and categorized according to Bandura’s framework as can be seen in the analysis within each sub-section.

### Participant Characteristics

All participants were working with MHAT. Most participants were women aged between 36 and 45 years, had completed secondary school (12 years of formal education, including kindergarten), and had up to 6 years’ experience in the field (see Table 2).

**Table 2:** Participants’ characteristics

Supervisees		Peer supervisors
	Number of participants (N = 12)	Number of participants (N = 2)
*Sex*		
Men	5	1
Women	7	1
*Age (in years)*		
25–35	1	
36–45	8	1
46–55	3	1
*No. of years of formal education*		
10	8	1
12	2	1
15	2	
*Experience in the field (years)*		
2–4	5	
4–6	6	
6–8	1	2

### Performance Outcomes

The first set of interview questions were directed at understanding how the peer supervision helped the supervisees in improving performance and developing a belief in their capacity to apply the necessary skills to achieve specific performance attainments as LMHWs. Based on the predetermined and emergent codes, performance outcomes were understood as consisting of improved quality of care, team work, self-care and administrative tasks.

#### Improving the Quality of Care

Quality care has a number of different characteristics and associated competencies. Within this study, specific topics and experiences emerged in which supervision allowed for improvements of practice. Aspects pertaining to the quality of care arising from the data are presented in Table 3, and discussed in the following paragraphs.

**Table 3:** Means of supervision and outcome in quality of care

Quality of care	Means of supervision	Qualities/learning outcomes
Preserving patients’ dignity	Corrective feedback, observation of the supervisor and verbal persuasion	Respecting patients’ time Keeping privacy and confidentiality Treating patients and caregivers with dignity as valued members
Provide accepted and relevant clinical and non-clinical care	Providing opportunity to observe how supervisor persuaded seeking help from other resources and sharing of their experiences	Ensuring social or family support Planning and organizing home visits in a community clinic Understanding receptiveness of the patient and family Accurate evaluation of emotional expressions and appropriate interpretation of the meaning of expressions Lack of adherence for follow-up Group facilitation Job-related issues Financial issues or other logistical issues Adherence to physical health treatment
Vocational rehabilitation	Providing opportunity to observe supervisor, support in seeking help from other resources and sharing of peer supervisors’ experiences	Assessing the patient’s needs Developing plans to meet identified needs Providing or arranging for the services the patient needs

##### Preserving Patients’ Dignity

The preservation of dignity during care is a fundamental component of evidence-based care and is highlighted in the LMHW classroom training curriculum. Nonetheless, the participants acknowledged that in practice some were initially less sensitive to this, to the extent that the peer supervisor needed to remind them. Study participants shared their experiences, where their peer supervisor was involved directly in their on-site supervision. The three means of support LMHWs experienced, as well as the specific points for maintaining dignity that they learned are demonstrated by the quotes discussed below. For instance, participant 7 discussed the effectiveness of receiving direct (corrective) feedback when she left a patient waiting too long.

My patient was waiting for 30 minutes for me in community clinic. That was noticed by my peer supervisor in on-site supervision. She gave feedback on this by reminding about the importance of respecting their time. (Participant 7)

Another means of learning was highlighted by participant 6 in the form of vicarious learning, noting that the peer supervisor intervened by commenting that they called patients into the consulting room without getting up from their desk and demonstrating how she herself went out to call patients in and greet them, explaining that this showed more respect. These actions correspond to the verbal persuasion and vicarious learning aspects of the SCP model for self-efficacy. Participants also linked this learning and improvement with better outcomes, for instance in the therapeutic relationship.

For the case of my client I apologized for making him wait after my peer supervisor’s feedback. The relationship after that was becoming warmer. (Participant 7)

Furthermore, dignity relating to treating patients and caregivers with value was highlighted by supervisors through correction that could be linked to the aspect of self-efficacy arising from the SCT framework, in the form of vicarious learning by observing an interaction between their peer supervisor and another community volunteer:

I noticed the way my peer supervisor corrected a community volunteer in the way a volunteer scolded [the] client for not having medication on time…. she corrected [the] volunteer by explaining that being seen as the person one is strengthens the experiences of value and a dignified life. (Participant 8)

Finally, privacy as an aspect of dignity was highlighted in participants’ experiences of supervision, where participant 8 noted that when encountering the supervisor outside the work environment, they advised the participant not to discuss patients, citing concerns about confidentiality.

##### Providing Accepted and Relevant Clinical and Non-clinical Care

This sub-theme emerged as a collection of specific skills related to care for which supervision provided guidance including continuity of care, understanding patients’ needs, scheduling activities, involving the family, and intervening in a timely manner to prevent relapse. Indeed, participants reported a significant improvement in the continuity of visits thanks to supervisors’ support on tackling delays or organizational problems. For instance, in cases where assessment and intervention were not resulting in progress in treatment, such that the participant was unsure of an appropriate course of action:

It was very difficult to move the sessions forward in a client who was bed-ridden for years without social or family support…discussed with clinicians, community volunteers and we couldn’t find any change at all. I was stuck...I realized that I am more confident in discussing with peer supervisor. I contacted my peer supervisor and I got opportunity to observe the session she does and try to replicate it. (Participant 6)

In this case it can be seen that observation, closely linked to the SCT point of vicarious learning, helped the participant to achieve self-efficacy. Supervisors also helped participants manage their own resources, enabling them to connect to other means of expertise, as in the following example:

In a psycho-education to the client and family, I faced difficulty […]. When I discussed this issue with [the] peer supervisor, she directed me to [the] concerned mental health professional. I sat with [the] peer supervisor and the mental health professional. They introduced the simple methods to understand the internal status of the client and family was very helpful in understanding clients, since these helped the clients to notice what was going on for them and to make sense of it. (Participant 10)

Supervisors were also able to foster new analytical skills among the participants in response to situations arising in treatment. For example, one participant illustrated how a change in analysis changed the patient’s response from the very first assessment by, for instance, accurately evaluating and interpreting emotional expressions. This trend of improved quality of care through supervision is further clearly visible in learning related to fostering the provision of social support, strengthening adherence to treatment, and timely intervention to prevent relapse. With regard to social support, for instance, while participants recognized the importance of involving the family in treatment, they often struggled initially with including them and were better able to do so after discussion with and observations of the peer supervisor.

Concerning treatment adherence, one participant in particular discovered how much could be gained from the knowledge and experience of their peer supervisor. Participant 6 gave the example of issues in scheduling activities for a patient with long-term depression, and was often unable to complete the tasks set. They sought advice from their supervisor who suggested setting more specific, measurable and achievable goals, which resulted in improved outcomes. Similar outcomes were experienced by participants 7, 10 and 11, each of whom learned about factors related to adherence and follow-up from their supervisor. Finally, participants described the benefits of peer supervision for identifying warning symptoms of relapse, which they considered to be a major aspect of providing quality care.

These encouraging findings notwithstanding, there were also some negative outcomes relating to communication with peer supervisors in this area. For instance, participants identified disagreement on conceptualization, diagnosis, treatment and theoretical orientation. This sometimes resulted in conflict for the participants in how to approach particular topics or issues concerning their patients. Furthermore, some participants encountered issues related to the time spent on supervision and indeed the quality of the supervision relating to outdated knowledge.

Does not spend supervision time supervising but instead chatting about various unrelated topics. (Participant 2)

She is not up with current theory and practice; very few clinical skills, etc. I only see her because I have to. (Participant 3)

These diverse responses occurred in the context of the participants’ characteristics that demonstrate the varied levels of experience, and therefore their different needs.

##### Vocational Rehabilitation

Support in the particular role of LMHWs in empowering clients in relation to returning to work came up with sufficient frequency to merit its own sub-theme related to the assessing of patient needs, planning on the basis of those needs and providing vocational rehabilitation and follow-up. As in previous examples, forms of support were identified as: observing the supervisor, identifying alternative sources of information, and sharing peer experiences. With regard to assessing patients’ needs, one participant noted the way their supervisor conducted initial questioning, in order to help the patient, identify a potential starting point:

[The] peer supervisor helped me in assessment stage by showing an assessment in this area and showed that how it helped my client to determine himself by asking […] What is the client’s present living condition? Is he dependent on someone else to provide basic services such as cooking and cleaning? Can he manage financial activities, such as handling an ATM card, opening a bank account, or living within a budget? […] This helped client to decide where to start. (Participant 10)

Study participants also explained how they received help from the supervisor in the second stage to develop a plan with a patient and what factors to consider. For example, the supervisors indicated that participants should consider the results of the assessments, detailed understanding of existing training resources in the patient’s community, understanding of employment opportunities in the local area, the feasibility of alternative goals when full-time employment is not an option, and how to empower the patient to make the necessary decisions. Finally, in the implementation phase supervisors helped increase knowledge about numerous forms of vocational assistance including supported employment, supported education, pre-vocational training and on-the-job training. The supervisors’ assistance in navigating these options is discussed in the following quotes:

Peer supervisor helps me to understand the vocational needs and how to address it with utilizing community as an asset. One of my clients[s] faced difficulty in getting [a] job. Then [the] peer supervisor showed me the ways how we can implement supported employment. Now that client is working in [a] nursery of [indoor] plants. (Participant 2)

One of my clients was interested in finishing tenth standard. [The supervisor] showed me how to do the supported education with the help of community clinic. My client took this as a new opportunity in her life and she said I never expected this. (Participant 5)

The peer supervisors helped the participants to connect with existing networks of employers and community volunteers, which gave them access to knowledge and resources. Participants explained how the peer supervisor guided them to access the community volunteers’ networks with employers and showed them how to tap into the knowledge of employment professionals. LMHWs and community volunteers can learn about which employers in the area train their new employees and on what terms, and then make helpful suggestions to their patients. For instance, as in the case of participant 10, the supervisor introduced knowledge on the way that practicing skills before entering explicit working conditions can be helpful for patients (Table 4).

**Table 4:** Areas and means of supervision and outcomes in improving teamwork

Areas	Means of supervision	Qualities/learning outcomes
Shared goals	Verbal persuasion	To deal effectively with colleagues who are working with them LMHW staff is having interpersonal issues with colleagues
Clearly defined roles	Verbal persuasion and sharing similar experiences which peer supervisor had gone through	Role confusion Procedures and process of decision making and implementation Lack of experience in organizational disciplines and procedures
Effective, timely communication	Verbal persuasion and sharing similar experiences which peer supervisor had gone through	Communication issues can occur within the team Communication with the patient
An optimistic, can-do attitude	Verbal persuasion to see importance in self; to reframe unhelpful beliefs; and to generate possibilities; and sharing similar experiences which peer supervisor had gone through	Complains about colleague’s negative attitude Always need reassurance Community clinic volunteers’ negative approach towards issues

### Improving Teamwork

Working as part of a clinical team is, as mentioned above, complex for LMHWs to navigate. As such, it is not surprising that teamwork emerged as a theme of peer supervision, where the supervisory relationship helped to understand key factors for success including: shared goals; clearly defined roles; shared knowledge and skills; effective and timely communication; and an optimistic, “can-do” attitude. Each of these is discussed with examples in the following sub-sections.

#### Shared Goals

While the setting of treatment goals in the clinical team as a part of care is covered in classroom training, this study found a gap in the articulation and following of these goals in practice. Community clinics exist within heterogenous and unique circumstances, and it is at times difficult to respond to a particular context without diverging from pre-established shared goals. One aspect of this complexity identified by the participants, in which supervisors supported them, was the tendency to try to please other colleagues, or other interpersonal issues with colleagues. This is clearly demonstrated in the following quotes:

When I discussed an issue between my colleague and me, my peer supervisor reminded me that our ultimate goal [,] is patient care. This helped me to ignore all other interpersonal or power issues in the way of our goal…patient care. (Participant 5)

In the initial period, I was focusing more impressing my colleagues through my outcome of sessions with clients. Unspoken, even unconscious goal was probably to impress the team. My peer supervisor noticed it and reminded me about the ultimate goal—that is patient care. (Participant 6)

These examples indicate the importance of verbal persuasion in supervision in relation to this outcome.

#### Clearly Defined Roles

Participants further described how they perceived the benefit of peer supervision in clarifying and defining their roles in each community and multidisciplinary team. In particular, given their embeddedness in the local context, it is at times difficult for LMHWs to distinguish their roles in the community clinic. Furthermore, providing the best care for patients also involves effective communication with different individuals and authorities, for which the division of responsibilities is also relevant. As such, the discussion and experience-sharing related to peer supervision proved useful for navigating uncertainty with respect to role divisions.

The procedures and process of decision making and implementation of [the] plan in each community clinic and with each mental health professionals are different. It is always anxiety provoking… Any smaller mistakes can cause larger impacts…so I usually share issue with my peer supervisor and get his opinions and experiences on similar and that gives me clarity in my role and others role… (Participant 10)

Interestingly, this lack of clarity on roles extends to an overall confusion about participants’ identity as LMHWs. This was largely related to a perceived lack of qualifications or authority:

I saw that [the] community clinic team were having issue in considering us as an LMHW staff of MHAT, and they have [an] issue in providing tasks to us. My peer supervisor is always help[ing] me to establish my identity. (Participant 12)

I was a housewife and community volunteer. I had no experience of previous jobs. I felt I needed to become skilled and establish the new identity as LMHW staff of MHAT like my peer supervisor. (Participant 5)

This theme emerged for 10 of the 12 participants, indicating its significance. Though this is most clearly an example of verbal persuasion and vicarious learning, it also shows the first clear example of a psycho-physiological response, which is discussed more under the heading of emotional support.

#### Effective, Timely Communication

In general, a number of participants found communication styles and practices and navigating these within clinical teams was a challenge, both in relation to interaction with the client and, for instance, the sharing of feedback in the team. For this reason, the supervisors’ input based on their own experiences, combining verbal persuasion and vicarious learning, made an important contribution for participants:

Any time any issue related to communication […] can happen within the team…so we need to be careful…if there is any doubt, we can contact our peer supervisor…this is really a help. (Participant 6)

If the communication with the client while intake is not [appropriate] there are chances of issues between [the] client and [the] treatment team. [The] peer supervisor reminds me [of] the importance of that communication all the time. (Participant 9)

#### An Optimistic, “can-do” Attitude

Finally, a more implicit code emerged in relation to the role of the participants’ attitude in how communication is received. Self-confidence, remaining positive toward clients and colleagues, and being respectful in engaging with the community all emerged as areas for improvement. On each of these, supervisors advised a strength-based approach by tackling self-confidence, reframing unhelpful beliefs, showing diverse possibilities and sharing their own experiences.

My supervisor always reassures me that I can do [it]. (Participant 11)

[The] peer supervisor teaches me to see the strengths of clients and families more than weaknesses and disabilities. (Participant 12)

Again, there is overlap with emotional support in the ways in which verbal persuasion is combined with a psycho-physiological response to strengthen the participants. In this aspect, however, it is worth noting that responses to supervisors were not uniformly positive as personality conflicts and communication issues also led to perceiving them as critical, judgmental or unsupportive, as one participant notes:

[My supervisor] is generally critical and not conscious of how her way of delivering peer supervision impacts my sessions and confidence. (Participant 2)

### Improving Self-care

It can be difficult to prioritize self-care when working in a new environment, and was especially so for participants who feared the impact of taking time for themselves on their clients. Peer supervisors were in a unique position to remind participants to care for themselves properly and avoid unnecessary health issues or burnout. This involved physical aspects of care like diet, exercise and monitoring of physical illness, mental aspects of self-care such as stress management, and emotional self-care relating to the general sense of wellbeing (Table 5).

My peer supervisor recommended [I see] a doctor when I was sick and taking the time to rest. He complimented [me] for caring [for] my physical health by eating extra fruit and veggies to fuel my body, as well as staying hydrated. (Participant 7)

My peer supervisor was encouraging my self-care that reduced unnecessary burnout by taking planned leave. (Participant 5)

As working experience is new to me, I felt difficulty in drawing [the] line. Sometimes I lost self-care. My peer supervisor helped me through sharing her experience. It was really helpful. (Participant 11)

**Table 5:** Areas and means of supervision and outcomes in improving self-care

Self-care	Means of supervision	Qualities/learning outcomes
Self-care (LMHWs’ health)	Verbal persuasion	Physical health
	Experience sharing	Preventing burnout
		Work–life balance

### Administrative Support

Though administrative tasks are generally covered in training, the practice of maintaining documentation and adhering to clinic policies was not routine among participants (Table 6).

**Table 6:** Areas and means of supervision and outcomes in administrative support

Administrative support	Means of supervision	Qualities/learning outcomes
Adherence to organizational policies	Monitoring and corrective feedback	Unplanned leave Policies
Documentation	Monitoring and corrective feedback	Language/spelling concerns

#### Improving Adherence to Disciplines and Policies

Adhering to policies is important for the functioning of an organization, for instance relating to managing leave, travel allowances and submission of reports. Supervisors identified gaps in these practices, especially in those with limited previous work experience, and responded accordingly.

It was difficult for me to follow the disciplines and policies. My peer supervisor taught me in supportive way and with corrective feedback. (Participant 12)

#### Documentation

Documentation was another area where participants reported lacking confidence. They expressed an intense fear of negative evaluation in writing, which led to procrastination. This was addressed by peer supervisors through carefully managed corrective feedback.

I was avoiding writing in the file…. very anxious about language, spelling etc. My peer supervisor taught me simple steps to overcome my fear. (Participant 4)

## Emotional Support

In relation to managing negative emotions that hinder work and learning processes, it made a difference to participants that there was someone on whom they could rely. Participants noted two forms of emotional support they gained from supervision: the creation of an accessible space to seek emotional support, and creating a motivating, positive environment (Table 7).

**Table 7:** Areas and means of supervision and outcomes in emotional support

Emotional support	Means of supervision	Qualities/learning outcomes
Availability and accessibility	Providing a secure space to discuss emotions	Addressing need for support
Emotional support	Creating a positive environment	Situational factors

### Availability and Accessibility

In relation to managing negative emotions that hinder work and learning processes, again it made a difference to participants that there was someone on whom they could rely. From their responses it can be gleaned that the availability of a peer supervisor as well as the manner in which the supervisor addressed them created a space in which they were well able to cope with emotions.

She gives me secure space for discussion when I need support. (Particpant6)

My peer supervisor is warm and nurturing. (Participant 12)

My peer supervisor is always available for me… that thought itself is relaxing for me… (Participant 10)

### Negative Emotional State Affecting Learning

Given that supervision also involves a certain degree of criticism, it is quite important that there is still drive or motivation to engage in the supervisory process. In general, participants expressed the view that the supervisors were able to create a positive space to engage with them. However, situational factors also play a significant role in motivation, for instance regarding the ways in which the supervisors themselves are treated within the organization. This was noted by one participant:

I felt bad in my peer supervisor’s approach in that community clinic’s issue. She received [a] negative outcome but didn’t realize that point…I feel less motivated in my sessions in my sessions with her. (Participant 2)

## Discussion

This discussion seeks to understand the application of peer supervision in one clinic in terms of its impact on self-efficacy and its consequences for performance outcomes, in order to generate insights for future application by identifying and evaluating challenges and their implications for future supervision.

This research was structured around the evaluation of a year-long peer-supervision program for LMHWs conducted in community clinics in Kerala. It successfully achieved the combined aims of evaluating the success of peer support in increasing self-efficacy and thus improving performance outcomes; identifying key challenges experienced by LMHWs; and will finally consider the implications of this data for future training and supervision. The findings of the qualitative data analysis on LMHWs’ perception of their peer supervision produced findings supporting the conclusion that peer supervision significantly enhanced self-efficacy, through an intricate and complex combination of supervisory means. This finding is consistent with that of Henry et al. (2016) and Hossain et al. (2021) who studied the effect of peer supervision of community health workers (CHWs) in improving quality assurance, communication and information, and creating a supportive environment. Furthermore, this research contributes to the literature on supervising LMHWs by identifying key challenges experienced by participants, which might be used to improve both classroom-based training and future supervision. For instance, while training covers such aspects as respecting a patient’s dignity, involving the patient’s family in treatment and other elements of performance discussed in supervision, the urgency of these issues had not been truly felt by participants until they were already working.

Concerning the fostering of self-efficacy through peer supervision, the central factors identified by Bandura (2008) were found to be consistent with our data on supervisory activity. The most directly visible form of this was the vicarious learning used by peer supervisors, where observing how they solved problems themselves served as a significant learning experience for participants—for instance, by showing a participant how to focus the assessment of socio-occupational functioning with the goal of achieving self-reliance. This resulted in the anticipated emotional development and sense of commitment (see Table 1). Similarly, supervisors used individual feedback and persuasion to guide their supervisees toward their own experiences of success. Where Bandura (2017) further notes the significance of positive feedback as supporting self-efficacy, this research built on this by showing the importance of building of a relationship of trust and of fostering a positive attitude. Furthermore, the role of self-care, physical and emotional wellbeing was emphasized just as strongly as in Bandura’s work. Overall, the findings showed that participants’ performance improved significantly in a manner corroborated in the literature on managerial strategies by Rogelberg (2017), showing that supervision can improve the employees’ belief in their capacities. Similar successful strategies have been found in relation to counselors and nurses in numerous studies (Augusta & Chandran, 2017; Bandura, 2020; Enlow et al., 2019; Morrison & Lent, 2018; Özteke Kozan, 2020; Vandament et al., 2021).

In terms of the challenges experienced by participants, these fell into four central categories: performance, team work, administrative and emotional support. Most of the research findings related to the first category, where each of the aspects identified concerned the significance of supervisory support in mediating the shift from theory or training to practice. For instance, it helped that supervisors emphasized the importance of dignity in high-quality care based on their practical experience, which is corroborated by a large body of literature, as this built on what had already been learnt in training (Dronet, 2016; Johnson et al., 2019; Kilbourne et al., 2018; Tomlinson, 2015). Of the other aspects of performance, it is possible to make a generalization that also forms the most interesting finding of this research: a large element of the support that the supervisors provided was passing on connectivity and understanding between the new LMHWs and the broader community. Whether in generating better social support for patients or discussing means of financial support and employment, supervisors consistently acted as mediators for creating connection. This finding presents an interesting answer to the research sub-question regarding the position of LMHWs in their communities. Indeed, collaboration with all concerned parties in a person’s treatment is essential, as is shared accountability (Uwisanze et al., 2021). The further evaluation of this finding is beyond the scope of this study, though in general, participants noted how collaboration and the transfer of skills from their supervisors made it easier to build skills and enhance their performance.

Performance did not, however, present the most significant challenge according to participants, who were more concerned by issues relating to team work and communication. Challenges related to setting boundaries, understanding distinct roles, and communicating in a timely and effective manner were noted across the board. Setting boundaries within multidisciplinary teams has previously been identified as a problem, for instance by Laurenzi et al. (2020). Interpersonal communication difficulties within teams, especially related to skill-sets and power dynamics, has also been identified in task-sharing situations (Ashengo et al., 2017). Concerning emotional support, which was identified in the literature as a significant element of mentorship and supervision, this study identified numerous areas of intervention. Managing stress, creating a sound work–life balance, and navigating difficulties and emergencies posed challenges for which participants turned to their supervisors. These results align with recent studies indicating that the relationship between the peer supervisors and the people they supervise is a key factor of effective supervision (Rothwell et al., 2021). This finding may be explained by the fact that the peer supervision included providing encouragement, rather than criticism or blame, when LMHWs were struggling. The importance of a positive and motivating form of supervision is also supported by recent findings (Bandura, 2020). Finally, administrative activities are those supervisory tasks which are required to keep a program running smoothly. The challenges observed in this area are mainly adherence to policies and absenteeism-related issues. These results support evidence from previous studies conducted among CHWs who were working in different areas of the health sector (O’Donovan et al., 2018; Ballard & Montgomery, 2017; Ballard et al., 2021). The administrative activities include knowing and effectively applying law and policy and ensuring compliance with deadlines and protocols regarding documentation. These activities facilitate efficient practice and serve to protect the organization, the staff and patients from costly mistakes.

On the basis of the data presented in this paper, some suggestions may be made for future work to address the needs and experiences of new LMHWs. First, the numerous instances in which information covered in training arose again within supervision indicates the need to address the gap between theory and practice in community health work. This provides a key argument for the future application of peer supervision more widely, given the need for experiential knowledge to address this. Second, issues relating to emotional support and self-care may indicate a potential gap in training, where the foundations for the necessary socio-emotional skill-set in such settings could be laid earlier on. Similarly, challenges with administration might be remedied by earlier attention to the importance of such tasks for smooth functioning. The question of LMHWs recognizing their roles and boundaries, as well as the significant connecting role of supervisors in the community, can be seen as further evidence of the importance of experiential support through peer supervision.

The research data also clearly shows how intricate and varied the supervisor’s strategies were in order to address all these different challenges LMHWs experience in the field. The intuition they showed in this research on effective supervision strategies were in line with other studies that identify local knowledge, collaboration and sensitivity to emotional needs as key factors (Avortri, Nabukalu & Nabyonga-Orem et al., 2019). This can be contrasted to the way in which financial, bureaucratic and political constraints can cause conventional supervisors to lack attentiveness to staff needs (Roberton et al., 2015). It is further noteworthy that in mental health services that are community-oriented and focused on wellbeing, rather than reducing mental illness, complexity quickly seeps in. The variety of skills, roles and support LMHWs are required to perform in such a context, exacerbated also by the low-resource setting, and issues of stigma and discrimination, supervision becomes even more cumbersome and abstract. The data presented in this study show that supervisors were quite well placed to guide and mentor their supervisees, reflecting a great degree of reflexivity, flexibility and responsiveness. As such future research might be more oriented towards understanding the backgrounds and experiences of supervisors, to discover how supervisors might be trained or supported in their work, especially as studies indicate that quality of supervision is of more significance than frequency. However, this study clearly shows the importance of flexibility and a level of presence (responding to the particular needs that are also embedded in the community context itself) in such complex settings as mental health care provision in low-income settings (e.g., Auschra, 2018).

## Limitations

It is important to make explicit and discuss the limitations of this study including: (a) the use of purposive sampling; (b) data from a single organization; (c) small sample size; (d) potential response bias; and (e) varying levels of experience with peer supervision among participants. Purposive sampling is a non-probability sampling technique that reduces the generalizability of the research findings (Crouse & Lowe, 2018; Jager et al., 2017) and increases the likelihood of selection bias (Crouse & Lowe, 2018). The small sample size could also present a limitation to the generalizability of these research findings to the specific study population (Vasileiou et al., 2018). Regarding internal validity and sample size, it is not known whether these research findings can account for the full field and variation of the phenomenon under investigation (Vasileiou et al., 2018). In addition to the limitations associated with a small sample size, it is possible that some participants exhibited response bias when answering the interview questions. For instance, participants might have responded in a way that is perceived as more desirable to the researcher (Villar, 2011). Moreover, other variables relating to the researcher’s demographic and interview characteristics could have potentially biased participants’ responses. Variables such as the researcher’s role in the organization could significantly facilitate bias in participant response (Villar, 2011). Finally, a variation in the degree of supervision received across the sample as well as the level of work experience was seen within the sample, and which may have implications for the self-efficacy of those LMHWs for whom the supervisor was not a good fit. Experience in community clinics might significantly influence LMHWs’ perceptions regarding the extent of the role supervision played in the initial development of clinical skill sets. A suggestion for future research, for instance in the form of realist evaluation, would be to include the perceptions and backgrounds of supervisors themselves, to complement the data of LMHWs.

## Conclusion

This study explored factors involved in implementing peer supervision in a community clinic in India, and its contribution to the development of LMHWs’ self-efficacy. It further catalogued challenges experienced by these LMHWs, and how these were tackled, as well as what the outcomes say about the role of LMHWs in their communities. Based on the manner in which supervision helped in dealing with difficult clients, identification and early resolution of issues arising in the community and issues related to discipline and organizational policy, it can be concluded that it made a valuable contribution to LMHWs’ self-efficacy. The research findings highlighted challenges related to communication, teamwork and applying learned behavior in real-life settings, and indicated that some of these issues are successfully tackled through peer supervision. Most interestingly, peer supervisors were also found to provide a necessary connection between new LMHWs and the communities and actors with which they need to collaborate to provide quality care. This study contributed to previous research by indicating the importance of the peer supervisors’ years of clinical experience and their skills and connections. This presents an interesting area for future exploration. This study further shows the practical implications involved in the application of peer supervision, demonstrating the usefulness of guidelines on structured supervision including accessibility, adherence to weekly schedules, and clear instructions for the supervisors.
